# Cardiac function and exercise capacity in patients with metabolic syndrome: A cross-sectional study

**DOI:** 10.3389/fcvm.2022.974802

**Published:** 2022-08-11

**Authors:** Jiming Chen, Xing Wang, Bin Dong, Chen Liu, Jingjing Zhao, Yugang Dong, Weihao Liang, Huiling Huang

**Affiliations:** ^1^Department of Cardiology, The First Affiliated Hospital, Sun Yat-sen University, Guangzhou, China; ^2^National Health Committee (NHC) Key Laboratory of Assisted Circulation, Sun Yat-sen University, Guangzhou, China

**Keywords:** metabolic syndrome, cardiac function, exercise capacity, impedance electrocardiogram, exercise tolerance test

## Abstract

**Background:**

Metabolic syndrome is a pre-diabetes condition that is associated with increased cardiovascular morbidity and mortality. We aimed to explore how exercise capacity, cardiac structure, and function were affected in patients with metabolic syndrome.

**Methods:**

Outpatients with echocardiography and exercise stress test combined with impedance cardiography (ETT + ICGG) results available from Nov 2018 to Oct 2020 were retrospectively enrolled. Echocardiographic, ETT + ICG profiles, and exercise performance were compared between patients with metabolic syndrome and the ones without. Sensitivity analyses were performed excluding patients without established coronary heart disease and further 1:1 paired for age and gender, respectively. Multiple linear regression was used to find out related predictors for maximal metabolic equivalents (METs).

**Results:**

Three hundred and twenty-third patients were included, among whom 97 were diagnosed as metabolic syndrome. Compared to patients without metabolic syndrome, echocardiography showed that patients with metabolic syndrome had a significantly lower E/A ratio (*p* < 0.001). Besides, they have larger left atrium, larger right ventricle, and thicker interventricular septum (all *p* < 0.001), but similar left ventricular ejection fraction (*P* = 0.443). ICG showed that patients with metabolic syndrome had significantly higher stroke volume at rest and maximum (*p* < 0.001), higher left cardiac work index at rest and maximum (*p* = 0.005), higher systemic vascular resistance (SVR) at rest (*p* < 0.001), but similar SVI (*p* = 0.888). During exercise, patients with metabolic syndrome had lower maximal METs (*p* < 0.001), and a higher proportion suffering from ST-segment depression during exercise (*p* = 0.009). Sensitivity analyses yielded similar results. As for the linear regression model, 6 independent variables (systolic blood pressure, BMI, E/A ratio, the height of O wave, the peak value of LCWi, and the baseline of SVR) had statistically significant effects on the maximal METs tested in exercise (*R* = 0.525, *R*^2^ = 0.246, *P* < 0.001).

**Conclusion:**

Patients with metabolic syndrome had significant structural alteration, apparent overburden of left ventricular work index, pre-and afterload, which may be the main cause of impaired exercise tolerance.

## Introduction

Since the conception of metabolic syndrome (MS) was described thoroughly in 2001 (1), MS has become a challenging problem due to its increasingly higher prevalence and cardiovascular disease-related morbidity (2, 3). MS is a state in which multiple metabolic risk factors of cardiovascular disease, including systemic hypertension, hyperlipidemia, and impaired fasting glucose, gather in individuals. As has been researched for years, MS is associated with many cardiovascular diseases, such as coronary heart disease, myocardial infarction, acute heart failure, cardiogenic shock, and so on (3).

In a previous study, clinical and subclinical systolic and diastolic dysfunction have been demonstrated by echocardiography in MS patients without coronary artery disease (4, 5). Furthermore, LV diastolic dysfunction, instead of systolic dysfunction, has been associated with limited exercise capacity independent of ischemia (6). However, some studies have come to different conclusions. Chung et al. indicated that the MS group had relatively high physical activity levels compared to the normal group of elderly women (7). So further studies are still needed for the evaluation of the exercise capacity and cardiac function of MS patients. In a previous study, the exercise capacity was assessed using echocardiography, exercise tolerance test, or cardiopulmonary exercise test, all of which were imaging or metabolic evaluation indicators (8).

Nowadays, impedance cardiography (ICG) can be used to record cardiac output continuously during exercise by combing with an exercise tolerance test, which provides us with another tool to evaluate the exercise capacity dynamically. High-definition impedance cardiography (HD-ICG), also known as signal morphology impedance cardiography (SM-ICG), is a radical improvement of ICG technology. Different from the conventional ICG, HD-ICG doesn't need to measure chest impedance (Z0) and chest geometric volume to evaluate baseline chest fluid. Accordingly, Obese, edematous, and moving patients can be measured much more accurately (9). In this study, we aim to explore how cardiac function and exercise capacity are affected in patients with MS, using echocardiography and high-definition impedance cardiography combined with exercise tolerance test (HD-ICG + ETT).

## Methods

### Study populations

In the present study, we retrospectively recruited non-heart failure patients who underwent HD-ICG+ ETT as well as echocardiography examination in Sun Yat-sen University's first affiliated hospital from January 2019 to October 2020. A total of 323 patients were recruited. Next, we excluded people who had been previously diagnosed with coronary heart disease and then matched MS patients and non-MS patients with gender and age as matching factors in remanent non-coronary heart disease patients (Specific matching methods were described later in statistical analysis).

### Diagnostic of metabolic syndrome

Metabolic syndrome (MS) was diagnosed following the National Cholesterol Education Program's Adult Treatment Panel III (NCEP: ATP III) criteria (1). Briefly, patients with at least 3 of the 5 conditions were diagnosed as MS: body mass index (BMI) ≥ 25 kg/m^2^, high blood pressure (≥130/85 mmHg), high triglyceride (≥150 mg/dL), low high intensity lipoprotein-cholesterol (HDL-c, <40 mg/dL for men and <50 mg/dL for women), and high glucose (≥110 mg/dL).

### Clinical measurement

At the beginning of the inspection, patients were required to undergo an evaluation of height and body weight, which were used for the calculation of BMI. Resting blood pressure, including systolic blood pressure (SBP) and diastolic blood pressure (DBP), were measured three times with an electronic sphygmomanometer, taking down systolic and diastolic values respectively. Patients would be recorded as “increased blood pressure” if they were recorded with resting systolic blood pressure ≥130 mmHg or diastolic blood pressure ≥85 mmHg. Besides, the history of hypertension was also recorded.

**Figure 1:**
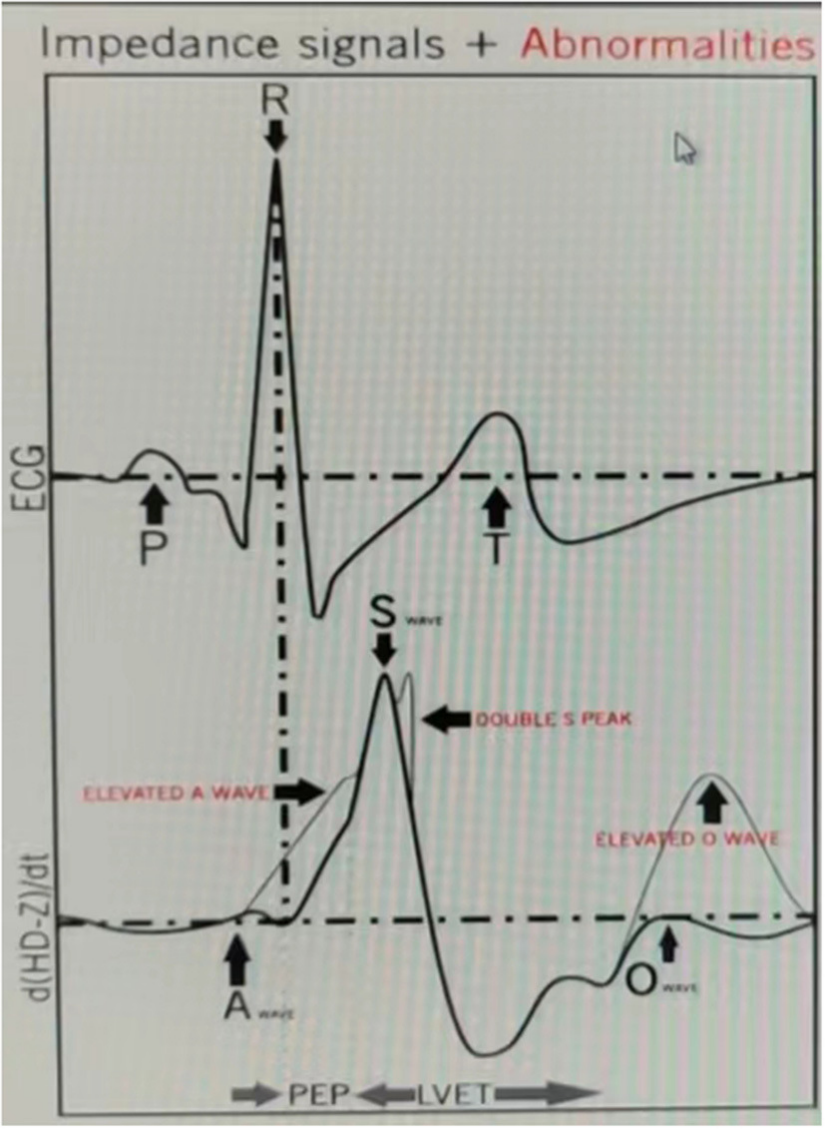
Impedance signals and the position of the A, S, and O wave.

Measurements included an M-mode echocardiogram performed after the selection of the measurement section by the B-mode scan. This allowed the assessment of left ventricular diastolic and systolic diameters, left ventricular ejection fraction (LVEF), left atrial diameter (LAD), right ventricular diameter (RVD), E/A ratio, E/E' ratio, and the thickness of each left ventricular wall, and stroke volume (SV) calculated by Simpson's method. The details about the acquisition of the A, S, and O waves are as follow in Figure 1. After a slight power delay, the P wave on ECG coincides with the first wave peak, that is A wave, in the second derivative waveform (dHD-Z/dt) which describes fluid acceleration, the A point. The A point marks the onset of late diastolic filling. The A wave appears only when there is atrial contraction, and its peak corresponds to the A wave of the Doppler echocardiogram. The second wave in the second derivative waveform (dHD-Z/dt) is the S wave, which helps evaluate cardiac contractility. The O wave represents the last wave in the second derivative waveform (dHD-Z/dt) and is associated with the opening of the mitral valve. The peak of O wave corresponds to the peak of the trans-mitral E wave detected by Doppler echocardiography.

Moreover, patients were able to undergo high-definition impedance cardiography with exercise tolerance test. Cyclometer was used to perform the exercise test. Wattage increments were determined by patient's age, gender, weight, and height. The test would be ceased until patients exhaust or ischemia-related symptoms occur. It could provide the height of A, S, O wave, stroke volume, stroke volume index (SVI), cardiac output (CO), cardiac contractility (CTI), left cardiac work index (LCWi), systemic vascular resistance (SVR), systemic vascular resistance index (SVRi), and other indicators at rest and exercise peak.

In addition, the type of SV change would be taken down and sorted into three types: (1) normal response meant that SV was elevated consistently during the exercise; (2) SV flat meant that there is a plateau during the exercise; (3) SV descent was described as a decline of stroke volume after a stable rise during the exercise. Meanwhile, the exercise tolerance test was assessed following the universal method, taking down the depth of ST-segment depression. ST-segment depression was also divided into three categories: (1) ST-segment depression within 0–0.1 mV was defined as no depression; (2) ST-segment depression within 0.1–0.2 mV; (3) ST-segment depression more than 0.2 mV was divided into two groups. In our study, we only extracted the details of each patient from our database while all the inspections were required in the diagnostic routine. Since it was an observational retrospective study, informed consent from participants was not needed.

### Statistical analysis

Continuous variables were presented as mean ± SD, and independent sample *t*-tests were used for comparisons between them. Categorical variables were presented as percentages and compared by chi-square tests. To further validate the differences in impedance cardiography and echocardiography between MS patients and non-MS patients, one sensitivity analysis was performed: (i) excluding those with coronary heart disease history (169 patients), (ii) further 1:1 paired with age (within 2 years old) and gender, in those without coronary heart disease history (154 patients). What's more, we used multiple linear regression to explore the relationship between metabolic equivalences and other indicators from echocardiography and impedance cardiography measured before. Besides, we used automatic linear modeling to explore the optimum combination of independent variables toward maximal METs. All statistical analyses were performed with SPSS version 26.0. *P* < 0.05 was considered statistically significant.

## Results

Clinical, demographic, echocardiography, and HD-ICG + ETT variables of the included patients and 1:1 paired patients were shown in Tables 1–4.

**Table 1:** The baseline characteristics of included patients and non-CHD one-one paired.

	**Included patients**			**Non-CHD one-one paired**		
	**Non-MS**	**MS**	***P***	**Non-MS**	**MS**	***P***
Age (years)	43.91 ± 15.83	51.37 ± 11.61	<0.001	48.78 ± 13.04	49.82 ± 11.38	0.599
Male	88, 38.94%	42, 43.30%	0.536	29, 37.7%	29, 37.7%	>0.999
BMI (kg/m^2^)	22.36 ± 2.91	26.00 ± 0.30	<0.001	22.60 ± 2.84	26.13 ± 3.08	<0.001
SBP base (mmHg)	118.42 ± 13.61	131.20 ± 16.18	<0.001	121.58 ± 14.61	132.03 ± 15.59	<0.001
DBP base (mmHg)	74.91 ± 9.34	83.99 ± 10.27	<0.001	75.96 ± 9.92	85.26 ± 10.56	<0.001
Hypertension/increased blood pressure	–	–	–	12(17.9%)	65(74.7%)	<0.001

CHD, coronary heart disease; MS, metabolic syndrome; BMI, body mass index; SBP, systolic blood pressure; DBP, diastolic blood pressure.

**Table 2:** The figures of ultrasound of included patients and non-CHD one-one paired.

	**Included patients**			**Non-CHD one-one paired**		
	**Non-MS**	**MS**	***P***	**Non-MS**	**MS**	***P***
LAD (mm)	30.89 ± 4.08	34.87 ± 3.83	<0.001	31.25 ± 4.14	34.60 ± 3.69	<0.001
RVD (mm)	20.66 ± 2.47	22.33 ± 2.45	<0.001	20.75 ± 2.38	22.40 ± 2.38	<0.001
IVS (mm)	8.72 ± 1.61	10.35 ± 1.66	<0.001	8.70 ± 1.57	10.34 ± 1.65	<0.001
LVEDD (mm)	45.58 ± 4.22	47.95 ± 4.83	<0.001	45.51 ± 3.56	47.71 ± 4.40	0.001
LVESD (mm)	27.53 ± 3.15	28.57 ± 4.02	0.026	27.22 ± 2.92	28.28 ± 3.76	0.052
LVPW (mm)	7.78 ± 1.25	9.23 ± 1.40	<0.001	7.81 ± 1.11	9.22 ± 1.43	<0.001
EF (%)	69.82 ± 5.48	70.67 ± 5.90	0.211	70.40 ± 5.28	71.05 ± 5.75	0.466
SV (ml)	66.96 ± 14.55	74.11 ± 16.14	<0.001	67.59 ± 12.40	74.03 ± 14.80	0.004
E	79.05 ± 20.05	69.42 ± 18.20	<0.001	76.29 ± 19.53	69.90 ± 19.15	0.042
A	66.02 ± 16.19	77.52 ± 18.08	<0.001	67.81 ± 15.83	76.91 ± 19.49	0.002
E/A	1.30 ± 0.46	0.96 ± 0.31	<0.001	1.20 ± 0.38	0.98 ± 0.32	<0.001
Average E/E'	7.57 ± 2.19	8.70 ± 2.73	<0.001	7.51 ± 1.76	8.58 ± 2.88	0.006
PASP (mmHg)	26.37 ± 5.18	26.79 ± 3.01	0.369	26.97 ± 6.45	26.88 ± 3.26	0.919

CHD, coronary heart disease; MS, metabolic syndrome; LAD, left atrium diameter; RVD, right ventricular diameter; IVS, interventricular septum thickness; LVEDD, left ventricular end-diastolic diameter; LVESD, left ventricular end-systolic diameter; LVPW, left ventricular posterior wall thickness; EF, ejection fraction; SV, stroke volume; PASP, pulmonary artery systolic pressure.

**Table 3:** The figures of high-definition impedance cardiography of included patients and non-CHD one-one paired.

	**Included patients**			**Non-CHD one-one paired**		
	**Non-MS**	**MS**	***P***	**Non-MS**	**MS**	***P***
Height of A wave	45.62 ± 25.75	46.47 ± 26.20	0.787	45.30 ± 26.37	48.40 ± 25.63	0.460
Height of S wave	219.88 ± 86.86	163.63 ± 71.04	<0.001	209.84 ± 87.32	164.97 ± 75.06	0.001
Height of O wave	21.38 ± 21.41	24.320 ± 18.17	0.238	21.95 ± 20.96	22.88 ± 17.86	0.767
SV base (ml)	65.96 ± 12.78	72.36 ± 11.27	<0.001	65.57 ± 12.73	71.40 ± 11.27	0.003
SV peak (ml)	93.75 ± 19.89	103.23 ± 16.99	<0.001	94.36 ± 20.49	102.88 ± 17.27	0.006
SV delta (ml)	27.79 ± 11.69	30.87 ± 10.44	0.026	28.79 ± 12.64	31.48 ± 10.40	0.151
SV delta percent	0.43 ± 0.17	0.43 ± 0.16	0.749	0.44 ± 0.19	0.45 ± 0.16	0.928
SVI base (ml/m^2^)	39.95 ± 7.27	40.73 ± 6.28	0.358	40.03 ± 7.69	39.99 ± 6.25	0.973
SVI peak (ml/m^2^)	58.704 ± 33.79	57.94 ± 9.95	0.827	56.70 ± 12.23	57.46 ± 9.94	0.672
SVI delta (ml/m^2^)	18.76 ± 32.15	17.21 ± 6.37	0.639	16.67 ± 7.25	17.47 ± 6.22	0.464
SVI delta percent	0.29 ± 0.09	0.29 ± 0.07	0.915	0.42 ± 0.17	0.44 ± 0.16	0.430
CO base (ml/)	5.18 ± 1.00	5.52 ± 0.98	0.005	5.11 ± 0.98	5.58 ± 0.98	0.004
CO peak (ml/)	13.92 ± 3.24	14.00 ± 3.03	0.835	13.73 ± 3.43	14.24 ± 3.15	0.335
CO delta (ml/)	8.73 ± 2.96	8.47 ± 2.68	0.457	8.62 ± 3.29	8.67 ± 2.78	0.924
CO delta percent (%)	0.62 ± 0.08	0.59 ± 0.09	0.038	1.75 ± 0.78	1.59 ± 0.52	0.128
EDFR base (%)	48.16 ± 8.95	51.17 ± 7.88	0.004	48.94 ± 8.13	51.09 ± 8.30	0.107
EDFR peak (%)	58.48 ± 17.28	62.78 ± 18.64	0.046	62.84 ± 18.94	63.30 ± 17.95	0.876
CTI base	223.65 ± 88.20	165.03 ± 69.44	<0.001	215.80 ± 92.38	166.65 ± 73.34	<0.001
CTI peak	377.54 ± 143.57	309.97 ± 127.45	<0.001	366.15 ± 156.76	308.87 ± 131.26	0.015
CTI delta	155.79 ± 104.14	146.56 ± 89.70	0.448	150.35 ± 112.39	142.22 ± 91.57	0.623
CTI delta percent (%)	0.23 ± 2.39	0.44 ± 0.19	0.421	0.78 ± 0.59	0.95 ± 0.69	0.096
LCWi base (kg·m/m^2^)	3.82 ± 0.92	4.27 ± 1.06	<0.001	3.95 ± 0.96	4.35 ± 1.05	0.016
LCWi peak (kg·m/m^2^)	10.824 ± 3.09	11.52 ± 3.76	0.085	10.73 ± 2.96	11.90 ± 3.81	0.035
LCWi delta (kg·m/m^2^)	7.01 ± 2.86	7.24 ± 3.31	0.512	6.78 ± 2.75	7.55 ± 3.40	0.121
LCWi delta percent (%)	0.63 ± 0.11	0.60 ± 0.13	0.106	1.79 ± 0.80	1.79 ± 0.80	0.981
VET base (ms)	395.61 ± 48.65	382.17 ± 45.79	0.021	399.21 ± 51.76	379.44 ± 45.13	0.013
VET peak (ms)	265.39 ± 51.50	258.01 ± 47.94	0.229	265.36 ± 51.35	255.67 ± 45.39	0.217
VET delta (ms)	130.22 ± 66.06	124.15 ± 63.73	0.445	133.85 ± 65.41	123.77 ± 60.47	0.322
VET delta percent (%)	0.32 ± 0.16	0.32 ± 0.14	0.863	0.33 ± 0.15	0.32 ± 0.14	0.753
SVR base (dyn·s/cm^5^)	2,271.95 ± 438.78	2,538.69 ± 411.82	<0.001	2,320.47 ± 514.95	2,547.09 ± 433.29	0.004
SVR peak (dyn·s/cm^5^)	1,054.92 ± 270.01	1,192.91 ± 272.89	<0.001	1,098.26 ± 310.21	1,180.99 ± 253.22	0.072
SVRi base (dyn·s/cm^5^·m^2^)	1,387.98 ± 272.27	1,418.46 ± 211.69	0.327	1,417.56 ± 310.40	1,416.03 ± 206.64	0.971
SVRi peak (dyn·s/cm^5^·m^2^)	641.84 ± 169.26	669.69 ± 143.03	0.157	670.86 ± 190.89	659.96 ± 136.55	0.684

CHD, coronary heart disease; MS, metabolic syndrome; SV, stroke volume; SVI, stroke volume index; CO, cardiac output; EDFR, end-diastolic filling rate; CTI, cardiac contractility index; LCWi, left cardiac work index; VET, ventricular ejection time; SVRi, systemic vascular resistance index; SVR, systemic vascular resistance.

**Table 4:** The distribution of SV change types and ST-segment depression of included patients and non-CHD one-one paired.

	**Included patients**			**Non-CHD one-one paired**		
	**Non-MS**	**MS**	***P***	**Non-MS**	**MS**	***P***
METs max	7.83 ± 1.64	6.81 ± 1.54	<0.001	7.52 ± 1.43	6.83 ± 1.41	0.003
SV change			0.894			0.344
Normal response	92, 40.7%	39, 40.2%		37, 48.1%	33, 42.9%	
SV flat	63, 27.9%	27, 27.8%		17, 22.1%	25, 32.5%	
SV descent	71, 31.4%	31, 32.0%		23, 29.9%	19, 24.7%	
ST-segment depression			0.022			0.012
No depression	188, 83.2%	81, 83.5%		72, 93.5%	59, 76.6%	
0.1–0.2 mV	22, 9.7%	10, 10.3%		3, 3.9%	13, 16.9%	
>0.2 mV	16, 7.1%	7, 7.2%		2, 2.6%	5, 6.5%	

CHD, coronary heart disease; MS, metabolic syndrome; METs max, maximal metabolic equivalences; SV, stroke volume.

### Among patients with and without metabolic syndrome

We found significantly higher BMI (*p* < 0.001), and systolic and diastolic blood pressure (*p* < 0.001) at rest in patients with MS. We conducted the comparison between MS and non-MS patients in echocardiography, which showed larger atrial and ventricular diameters and thicker ventricular thickness in patients with MS, but no difference in EF (*p* = 0.211), and pulmonary artery systolic pressure (PASP) (*p* = 0.369). As for behaviors in Impedance cardiography, some systolic variables were significantly different, including the height of S wave (*p* < 0.001), SV base and peak (*p* < 0.001), CO base (*p* = 0.005), CTI base and peak (*p* <0.001), LCWi base (*p* < 0.001), and ventricular ejection time (VET) base (*p* = 0.021). Other systolic variables were not significantly different. In terms of diastolic variables, early diastolic filling rate (EDFR) base (*p* = 0.004), SVR base (*p* < 0.001), and SVR peak (*p* < 0.001) in patients with MS were significantly higher than in patients without. In addition, the EDFR peak (*p* = 0.046) in patients with MS seemed to be higher than in patients without. There is no difference in SV change between the two groups. The proportion of ST-segment depression seemed to be lower in patients without MS, while maximal METs were higher in patients without MS.

### Among patients without coronary heart disease with 1:1 paired

For studying the differences further, we did a sensitivity analysis. As shown in Table 2, compared with non-MS patients, patients with MS were not significantly older (*p* = 0.599), but had a higher BMI (*p* < 0.001) as well as higher blood pressure (*p* < 0.001) at rest. For the echocardiographic parameters, MS patients showed noticeable myocardial hypertrophy [thicker LVPW (*P* < 0.001) and IVS (*P* < 0.001)] and cardiac enlargement [LAD (*P* < 0.001), larger RVD (*P* < 0.001) and larger LVEDD (*P* = 0.001)], as well as significantly worse diastolic function [lower E/A ratio (*P* < 0.001) and higher E/E' ratio (*P* = 0.006)], but similar LVESD (*p* = 0.052) and EF (*p* = 0.466).

Functional data were measured by impedance cardiography between patients with and without MS. It could be found that SV (*P* = 0.003) and CO (*P* = 0.003) in patients with MS were significantly higher than in those without MS under rest state. Moreover, lower height of S wave (*P* = 0.003), shorter left ventricular ejection time (*P* = 0.013), lower cardiac contractility index (CTI) (*P* < 0.001), higher left cardiac work index (LCWi) (*P* < 0.001) and higher systemic vascular resistance (SVR) (*P* = 0.004) were found in MS patients at rest. No significant difference was found in SVI (*p* = 0.430), EDFR (*P* = 0.107), and systemic vascular resistance index (*P* = 0.971). While comparing the indicators at peak exercise, patients with MS seemed to have higher SV (*P* = 0.006), lower CTI (*P* = 0.015), and higher LCWi (*P* = 0.035). No difference was found in other indicators when patients were at peak exercise. Besides, we also used the difference, which equaled the value at peak exercise minus the value at rest, to compare the trends of all the indicators between MS and non-MS patients from rest to peak exercise. As for the result shown in Table 3, it seemed that patients with and without MS have the same trends in the indicators included.

During ETT, we didn't find any difference in the frequency of SV change (*P* = 0.344) between the patients with and without MS. However, we noticed that the proportion of ST-segment depression (*P* = 0.012) was higher in patients with MS. Besides, patients with MS showed significantly lower maximal METs (*P* = 0.001) during ETT.

**Figure 2:**
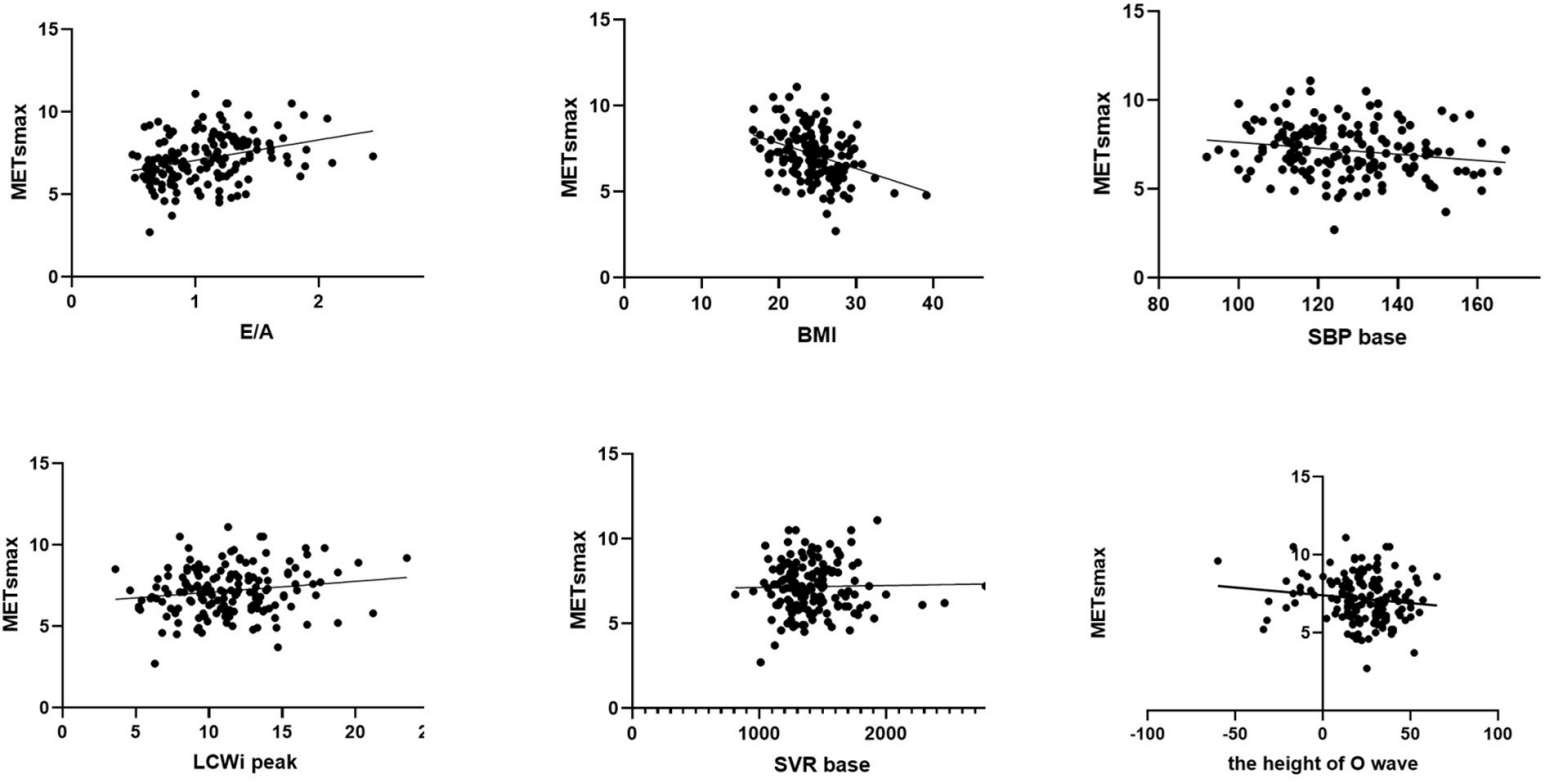
Scatterplot of simple linear regression equation of E/A ratio, BMI, SBP at baseline, LCWi at peak, SVR at baseline, the height of O wave, and Maximal METs, respectively.

Afterward, we used automatic linear modeling to find out the optimum assembly of independent variables toward maximal Mets. It showed that among the indicators included, the best subset was the pattern of hypertension/increased blood pressure, BMI, E/A, LCWi peak, and SVR base. In Figure 2, the linear regression of each independent variable with METs was displayed. Meanwhile, we performed scatterplots to recognize the relationship between each variable and METs shown in Figure 2. It showed that the E/A ratio and LCWi peak have a positive correlation with METs while BMI, SBP base, and the height of the O wave were negatively correlated with METs. Perhaps oddly, it seemed that the correlation between SVR base and METs was too weak to be displayed in the figure. Then we made a linear regression model to explain the relationship between the pattern above and METs. Thereafter we found a formula as follows:


$$\begin{array}{l} \text{METs }=\;6.922-0.707\text{H}-0.105\text{B}+0.697\text{E}-0.014\text{O}\\ \;+\;0.102\text{L}+0.001\text{S} \end{array}$$

(H means hypertension/increased blood pressure; B means BMI, E means E/A ratio; O means the height of O wave; L means LCWi peak; S means SVR base.)

According to Table 5, the formula is of significance (*P* < 0.001) while its explanation for METs was a little weaker (*R* = 0.525, *R*^2^ = 0.275).

**Table 5:** The multiple linear regression results between METs max and the independent variables below.

	**β**	**Standardized coefficient β**	***P***	***R***	***R^2^***	**Adjusted *R*^2^**
Equation			<0.001	0.525	0.275	0.246
Constant	6.922	–	<0.001			
Hypertension/pre-hypertension	−0.707	−0.242	0.006			
BMI	−0.105	−0.248	0.002			
E/A ratios	0.697	0.178	0.028			
The height of O wave	−0.014	−0.184	0.012			
LCWi peak	0.102	0.242	0.002			
SVR base	0.001	0.221	0.004			

BMI, body mass index; LCWi peak, the peak value of left cardiac work index; SVRi base, systemic vascular resistance index at rest; METs max, maximal metabolic equivalents.

## Discussion

The main results of this study, in which we have assessed the differences in cardiography and impedance cardiology with exercise tolerance test between patients with and without MS after excluding coronary heart disease, were: (1) to find subclinical cardiac hypertrophy, left ventricular diastolic dysfunction (LVDD). (2) to discover higher cardiac work and systemic vascular resistance. (3) to explore possible equations to predict the metabolic equivalents.

### Structural differences in echocardiography

We used echocardiography to evaluate the structural changes in patients with MS but without CHD. Compared to the group without MS, patients with MS would show subclinical ventricular hypertrophy and chambers enlargement while the result was consistent with previous observational studies (10, 11). The ventricular hypertrophy seemed to be the product of MS, being associated with higher SV and cardiac work at rest. Even though LVEDD in MS patients was apparently higher, the left ventricular ejection fraction (LVEF) between the two groups was similar. It can partly be explained that the increment of LVEDD in MS patients was negligible as it was proved that clinical systolic dysfunction usually happens after diastolic dysfunction (12) while Nir Ayalon's research suggested that subclinical left ventricular diastolic dysfunction may be associated with MS but not left ventricular hypertrophy (10). Left atrial enlargement, which marks the structural change of the left heart, has been proved as an independent predictor of exercise capacity in patients with isolated diastolic dysfunction presented with exertional dyspnea (13).

### Functional differences in echocardiography and impedance cardiography with exercise tolerance test

While routine echocardiography can only measure hemodynamic at rest, HD-ICG is a technique with kinetic consistent hemodynamic measurement, which helps analyse the change of cardiac function during exercise. More importantly, bioelectric impedance has been proved to have prognostic value in patients with heart failure (14), further research had been done to report that ICG responses during exercise offer important reclassification for predicting risk for adverse outcomes in heart failure (15, 16). As is known, the E/A ratio and E/E' ratio are reliable indicators for diastolic dysfunction. Differences in these two parameters, which presented subclinical diastolic dysfunction of LV in patients with MS, were in line with previous studies (7, 17, 18). The deterioration of diastolic function was also consistent with left heart structure alterations in the research. Moreover, it was reported that diastolic dysfunction increased the incidence of cardiovascular events or death by 2.53 times (19). Therefore, detection of subclinical diastolic dysfunction in MS patients seems reasonable.

In terms of systolic function, higher stroke volume and similar LVEF were caught in MS patients through echocardiography. Similarly, Grandi et al. (20) reported that only left ventricular diastolic dysfunction was detected in metabolic syndrome except for left ventricular systolic function (LVSF), which was also parallel to the research of Masugata et al. (12). All these parameters above were measured at rest. Besides, we also used HD-ICG + ETT to evaluate hemodynamics and exercise capacity during incremental exercise, in which higher output status was recorded both at rest and exercise peak. Even though SV at both time points was significantly higher in MS patients, the stroke volume index was similar to the control group at both time points. On the one hand, it can partly attribute to higher body surface area (BSA) and BMI. On the other hand, cardiac contractility (CTI) in MS patients was recorded as lower than in the controlled. It seemingly implied that though MS patients showed higher SV, their SVI resembled the normal. Nevertheless, the worse CTI discovered in MS patients suggested that this group may have dysfunction in cardiomyocyte contractility. Hence, we considered that it indicates the contractility deterioration at the cardiomyocyte level, which may be associated with myocardial hypertrophy. In addition, it complied with the opinion of van Heerebeek et al. that insisted that myocyte stiffness instead of increased fibrosis was the main explanation for cardiovascular characteristics in MS (21). Meanwhile, the S wave was lower in MS patients than in non-MS patients. As is known, the S wave indicates cardiac contractility. We also noticed a decreased CTI in MS patients. The change of S wave corresponded to CTI, which also coincides with the identified order of cardiac dysfunction in patients with MS (12).

### Exercise capacity and cardiac dysfunction

In our study, we evaluated exercise capacity using metabolic equivalents in exercise tolerance test. Our results were in line with previous studies that showed lower exercise capacity in patients with arterial hypertension, especially in those females with dyspnea (22, 23). A previous study evaluated the exercise capacity between the pre-hypertension group and the normal-blood pressure group, however, the result showed that no difference was found between them though subclinical cardiac structural and functional changes truly existed, such as increased left ventricular mass index and diastolic dysfunction (24). Lower exercise capacity was proved to be associated with MS in patients with coronary heart disease while using heart rate recovery (HRR) to assess the sympathetic balance index (25). In our study, it was found that compared to NMS patients, MS patients without CHD were still along with lower exercise capacity, which implied that MS directly influences the exercise capacity of individuals. SVI is an SV index that excludes the effect of height and weight, which was comparable between the two groups. It seems as if the similar SVI in two groups can lead to the conclusion that the systolic function of the LV is intact. Nevertheless, patients with MS had lower maximal metabolic equivalents during the exercise, which may deduce that diastolic dysfunction may be involved in the impairment of the exercise capacity. Besides, patients with MS had significantly higher systemic vascular resistance index, which might be related to the high prevalence of hypertension in MS.

### HD-ICG with ETT and cardiac dysfunction

Impedance Cardiography (ICG) is a non-invasive and unobtrusive technique for measuring cardiac output. It assesses instant changes in thoracic electrical impedance to calculate hemodynamic variables and provides a way to dynamically evaluate SV changes. What's more, the sensitivity, specificity, and positive and negative predictive values for ICG were 100, 50, 79, and 100%, respectively, with coronarography as a gold standard for comparison (26). It is known that in the myocardial ischemia model, the reduction or loss of localized ventricular wall motion occurs firstly, followed by abnormal ST-segment changes and later pain and related symptoms. That is to say, there is an asymptomatic period in the process of myocardial ischemia, from the onset of imbalance between oxygen supply and oxygen demand to the clinical appearance of painful symptoms, which is called the “ischemic gap.” In the included patients with metabolic syndrome, myocardial ischemic-related painful symptoms were not reported. However, it was noteworthy that recorded ST-segment depression in the metabolic syndrome group was more than in the non-metabolic syndrome group during submaximal exercise using ICG + ETT. The above phenomenon indicated that it is counterbalanced between myocardial oxygen supply and oxygen demand in patients with metabolic syndrome at rest while oxygen demand is far beyond the oxygen supply during strenuous exercise. It may imply that though no apparent stenosis was found in the main vessels, there may exist stenosis in micro vessels. Therefore, it is more direct to observe whether coronary microcirculation ischemia exists by dynamically measuring ICG + ETT-related data. Further research is needed to prove the hypothesis. Besides, the proportion of ST-segment depression was significantly higher in patients with MS, while there was no significant difference in SV increment between patients with and without MS. Pre-hypertension and central obesity may involve in the progression of myocardial ischemia since hypertension can accelerate arteriosclerosis by forcing endothelial cells and arterial smooth muscle cells to be chronically exposed to increased dilatability of the arterial wall (11). Whereby SV increment didn't change alongside with ST-segment, we speculate that the impairment of the exercise capacity might have nothing to do with obstructive coronary artery disease, which may result from functional myocardial ischemia, caused by increased end-diastolic pressure of LV as a result of diastolic dysfunction. The speculation needs further investigation to be proved. Furthermore, a large proportion of SV plateaus or decreases were noticed. However, the presence of SV plateaus or decreases in the normal population may also be a physiological response. A previous study has shown that echocardiography immediately after endurance exercise reveals a mild decrease in diastolic function with or without a decrease in systolic function, which some scholars refer to as “cardiac fatigue.” This indicated that in the non-MS population, transient cardiac dysfunction occurs when the exercise load during HD-ICG + ETT exceeds its extreme limits. Of course, comparative studies need to be refined to further confirm the hypothesis.

As for the multiple linear regression model, six independent variables were involved in the equation to determine individuals' maximal metabolic equivalents (METs). METS is an index reflecting relative energy metabolism level and exercise intensity. For patients with neither lung diseases nor musculoskeletal diseases, the connection between E/A ratio, systolic blood pressure, diastolic blood pressure, and METS can be explained as the impairment of the exercise capacity caused by diastolic dysfunction and hypertension. This is consistent with previous studies (6, 27–30). Notwithstanding, the *R*^2^, which is used to analyze the explanatory power of the equation, showed that there was a slightly weak correlation between the two sides of the equation. Hence, the formula may not explain the maximal METs well, implying that its prediction was not strong enough. More research should be done to figure out better equations for METs.

## Limitation

This study has some limitations. Firstly, since our research is a cross-sectional study, longitudinal and/or interventional studies are needed to further confirm our hypotheses. Secondly, our study tried to rule out patients with coronary heart disease, but not all the patients had coronary angiography examination, and the existence of coronary heart disease can't be totally ruled out by symptoms, Holter, and transthoracic echocardiography. Thirdly, although previous articles comparing ICG with ultrasound have demonstrated that there is no significant difference between the data obtained by ICG and ultrasound, and that the measurement error of HD-ICG is minimal due to its high accuracy, there may still be some difference with the actual SV value based on the algorithm problem of ICG itself for the SV value. Therefore, the calculation method of SV value should be continuously improved. Fourthly, more comparative analyses, which take age, gender, hypertension, smoking status, diabetes, and other risk factors of coronary diseases into account, should be done to find more details. Last but not least, we interpret the ST-segment depression as functional myocardial ischemia, caused by diastolic dysfunction due to a pre-hypertension state. This speculation needs further examination to be confirmed.

## Conclusion

From the results we've got above, Patients with Mets showed significant structural alterations. Besides, noteworthy diastolic dysfunction was observed in patients with MS. The structural alterations and diastolic dysfunction may be the main cause of impaired exercise tolerance. Therefore, further research needs to be done to verify the relationship between exercise capacity and metabolic syndrome, which may help formulate the management of MS.

## Data availability statement

The original contributions presented in the study are included in the article/supplementary material, further inquiries can be directed to the corresponding authors.

## Ethics statement

The studies involving human participants were reviewed and approved by Ethics Committee of the Department of Scientific Research, The First Hospital of Sun Yat-sen University, Guangzhou, China. Written informed consent for participation was not required for this study in accordance with the national legislation and the institutional requirements.

## Author contributions

HH and XW conceived and designed the review. JC, BD, and WL collected the data and contributed to the analysis of literature data. WL and JC performed the analysis of all data. XW, JC, BD, CL, JZ, YD, WL, and HH all participated in the discussion of the results. XW and JC wrote the paper. All authors contributed to the article and approved the submitted version.

## Funding

This study was funded by the Guangdong Province Natural Science Foundation (2021A1515010114) and State Key Laboratory of Organ Failure Research, Southern Medical University, Guangzhou, China (Item No. 202002).

## Conflict of interest

The authors declare that the research was conducted in the absence of any commercial or financial relationships that could be construed as a potential conflict of interest.

## Publisher's note

All claims expressed in this article are solely those of the authors and do not necessarily represent those of their affiliated organizations, or those of the publisher, the editors and the reviewers. Any product that may be evaluated in this article, or claim that may be made by its manufacturer, is not guaranteed or endorsed by the publisher.
